# Report of the Cases and Operations Which Have Occurred in the Surgical Wards of the Pennsylvania Hospital, during the Service of Dr. Thomas Harris, (the Months of November, December, January, and February)

**Published:** 1839-03-23

**Authors:** H. H. Smith

**Affiliations:** Resident Surgeon


### MEDICAL EXAMINER.

DEVOTED TO MEDICINE, SURGERY, AND THE COLLATERAL SCIENCES.

No. 12.] PHILADELPHIA, SATURDAY, MARCH 23, 1839. [Vol. II.

REPORT *of the Cases and Operations which have occurred in the Surgical Wards of the Pennsylvania Hospital, during the service of* Dr. Thomas Harris, (*the months of November, December, January, and February.*)

[Reported by H. H. Smith, M. D., Resident Surgeon.]

The Pennsylvania Hospital is an institution established principally by private contributions, and receives four classes of persons.

*First.* Sailors belonging to the merchant service, who may require medical attendance, and for whom a certain sum is paid by the custom-house, out of a fund accumulated by the monthly contributions of each American sailor.

*Second.* Respectable poor individuals, labouring under surgical or medical diseases, except syphilis, or incurable, or contagious affections.

*Third.* The insane.

*Fourth.* Any one, who may be accidentally injured, and brought to the hospital within twenty-four hours after the occurrence.

The buildings consist of a centre, sixty feet front; of two wings, each eighty feet long, by twenty-seven broad; and of two return wings, one hundred and ten feet in depth;—the whole of which will accommodate two hundred and fifty persons.

They are situated on a square of ground containing about four acres, neatly laid out, and affording opportunity for free ventilation and exercise. The whole area is surrounded by a wall, and by rows of noble forest trees.

The centre is built in the old style, with wide halls and stairways, lofty ceilings, &c., and contains in the third story an operating-room, lighted from the roof, and capable of holding two hundred and fifty students; a long ward, fifty feet by twenty-three, containing twelve beds appropriated to the female surgical cases; two smaller rooms, with four beds each, for cases operated on, or otherwise important; and on the first floor a medical library of near eight thousand volumes, which is supported by the fees of the students attending the practice of the hospital.

On the west of this, the wing is occupied entirely by the insane,—and on the east, in the first story, is the ward appropriated to the male surgical cases, which, as well as the return wing, accommodates forty beds. The rest is occupied by the male medical cases, numbering somewhat less. Near to this end is a distinct building occupied by the syphilitic sailors and the blacks, which contains thirty-five beds, making the whole number in charge of the surgeon one hundred and five.

The wards are warmed by furnaces of anthracite coal, placed in the cellar; they are well lighted by windows facing north and south, and are ventilated by openings into the chimnies near the ceilings—the warm air always securing a constant draught.

In the surgical wards, the bedsteads are made of iron, with wire nettings for the beds,—and in those appropriated to fractures, there is a frame underneath, in which a pan slides, with a corresponding hole in the hair mattress above, so that it is never necessary for the patient to move in the least from the perfectly supine posture.

The dressings are such as are readily applied, and economical in their use. The ground flax-seed cake, mixed with warm water, and slightly oiled on the surface, constitutes the poultice, and answers quite as well as the bread and milk, while it is also less expensive, a very necessary consideration in all hospital dressings.

The splints and apparatus for fractures are such as have been reported by Dr. J. M. Wallace, in No. 2, vol. I., of the Examiner, except that within the last two months the *immoveable apparatus* has been in use.

The duties of the house are divided among three surgeons—Drs. Thos. Harris, Randolph, and Norris; and three physicians—Drs. B. H. Coates, Wood, and Stewardson; each of whom serves four months, and visits the patients at least twice a week, at which time clinical instruction is given. There are, also, during the winter months, clinical lectures delivered weekly.

The daily visit is made by two residents, graduates in medicine, who take charge of the wards alternately, and serve two years from the period of appointment.

The annexed account of cases contains only those admitted during November, December, January, and February, under the charge of Dr. Harris. Those cases, therefore, which are recorded as having been operated upon, but which are not included in the list of admissions, consist of those which were in the hospital at the commencement of his term. Of the whole number of patients, a large proportion will be found under the head of fractures and contusions, the numerous buildings, quarries, &c., in the neighbourhood, giving rise to many such injuries. Some slight difference will also be found between the number admitted and the number of diseases, as many patients labour under more than one affection at the time of entrance, as burns, lacerations, and fractures, from blasting, &c., while the more serious one only is noted.

At the commencement of the season there were seventy patients in the wards, since which time one hundred and forty-two have been admitted, of whom one hundred and twenty-six have been discharged, and thirteen have died, leaving seventy-three at present in the institution.

The following tables will show the diseases admitted, and the success of the treatment.

*List of Cases treated.*

| | |
|---|---|
| *Abscess* | 1 |
| " of the hip | 1 |
| *Amaurosis* | 1 |
| *Buboes* | 5 |
| *Bullæ* | 1 |
| *Burns* | 4 |
| *Calculus in the bladder* | 1 |
| *Concussion of the brain* | 2 |
| *Contusions* | 13 |
| *Curvature of the spine* | 2 |
| *Diseased bladder* | 3 |
| *Dislocations* | 2 |
| " of the hip | 1 |
| " of the elbow | 1 |
| *Ecthyma* | 1 |
| *Erysipelas* | 2 |
| *Fistulæ* | 3 |
| " lachrymalis | 1 |
| " in perineo | 1 |
| " in ano | 1 |
| *Fractures* | 39 |
| " skull, compound | 3 |
| " clavicle | 1 |
| " humerus, compound | 1 |
| " " simple | 4 |
| " radius and ulna, compound | 1 |
| " " " simple | 1 |
| " ulna, simple | 1 |
| " thigh, compound | 2 |
| " " simple | 7 |
| " leg, compound | 4 |
| " " simple | 8 |
| " " fibula | 4 |
| " ribs | 2 |
| *Frost bite* | 1 |
| *Gonorrhœa* | 5 |
| *Hemorrhoids* | 1 |
| *Hernia* | 5 |
| " inguinal, reducible | 2 |
| " femoral, strangulated | 1 |
| " cerebri | 2 |
| *Herpes* | 2 |
| *Hydrops articuli* | 2 |
| *Ophthalmia* | 12 |
| " opacity of cornea | 1 |
| " iritis | 2 |
| " conjunctivitis | 4 |
| " pterygia | 2 |
| " cataract | 2 |
| " sclerotitis | 1 |
| *Orchitis* | 4 |
| *Amputations* | 7 |
| " thigh | 3 |
| " leg | 1 |
| " arm | 2 |
| " finger | 1 |
| *Cataract* | 5 |
| *Caries sterni* | 1 |
| *Erectile tumour* | 1 |
| *Extirpation of parotid*, (by Dr. Randolph) | 1 |
| *Fistula lachrymalis* | 1 |
| *Hydrocele* | 1 |
| *Hemorrhoids* | 1 |
| *Hernia, strangulated* | 1 |
| *Necrosis* | 3 |
| *Phymosis* | 1 |
| *Pterygia* | 1 |
| *Stone in bladder*, (lithotomy) | 1 |
| *Trephining* | 1 |
| *Varicose veins* | 1 |
| *Porrigo capitis* | 1 |
| *Pterygia* | 2 |
| *Phymosis* | 1 |
| *Paraphymosis* | 2 |
| *Sprains* | 3 |
| " ankle | 3 |
| *Scirrhus* | 1 |
| " parotid | 1 |
| *Scrofulous abscesses and tumours* | 3 |
| *Syphilis* | 14 |
| *Stricture of urethra* | 2 |
| *Ulcers* | 16 |
| *Varicose veins* | 2 |
| *Wounds* | 15 |
| " *incised*, of throat | 1 |
| " " of wrist-joint | 1 |
| " " of knee-joint | 1 |
| " " of head | 1 |
| " " of face and tongue | 1 |
| " *lacerated*, of hand | 1 |
| " " of thigh | 3 |
| " " of leg | 3 |
| " " of scalp | 2 |
| " " of eyelids | 1 |

Of the above, thirteen have died, viz:

| Disease. | No. | Remarks. |
|---|---|---|
| *Burns* | 2 | The whole of the body; death within 24 hours after admission. |
| *Concussion of brain* | 1 | Never free from stupor. |
| *Fractures* | 7 | |
| " thigh, compound | 2 | Both within 48 hours, being complicated with other injuries. |
| " leg, comp'nd | 1 | Of mortification. |
| " skull, " | 2 | |
| " leg, simple | 2 | One of mortification, and one of mania-a-potu. |
| *Incised throat* | 1 | Mania-a-potu following. |
| *Hernia cerebri* | 2 | |

*Fractures.*—This class of injuries constitutes the great mass of our recent cases. On the receipt of a case of the lower extremity, the settee on which the patient is brought is placed alongside of the fracture-bed, prepared as mentioned, the clothes are carefully removed, the patient perfectly cleansed, and then by putting the settee close to the bed he is readily transferred. If the fracture is of one or both bones of the leg, it is placed in the fracture-box, protected by oil cloth to allow of cooling applications; if of the thigh, in Physick's modification of Desault's long splints, most frequently without the bandage of Scultetus, that cold applications may be made directly to the part, and the adjustment of the fracture may be accurately observed. This latter dressing is that generally used for the thigh; but, owing to the prevalence of erysipelas, sloughing of the heel from the extending band has so often happened, that the long fracture-box, attached to a double inclined plane, has been substituted during the winter, the sides of the box affording support to the thigh, and the weight of it preventing any variation from the original position of the limb. Several cases have lately been treated by the *immoveable apparatus* with perfect success. This apparatus has obviated the danger of sloughing of the heel in fractures of the thigh, by doing away with the extending band, and also relieves the patient from the risk of a slough on the sacrum, by allowing him to walk about soon after the injury. It also answers an excellent purpose in the treatment of fractures of the leg and its complications, as by the erect posture the patient's health is preserved, and callus is more quickly deposited than if he were confined to bed. In the mania-a-potu, which very frequently supervenes on these accidents, it also prevents any displacement of the fracture or injury of the soft parts. As an account of the mode of application of this apparatus, with cases in detail, has been published in a previous number, it is unnecessary now further to advert to the subject.

*Erysipelas.*—This terrible scourge of hospitals has prevailed very frequently throughout the institution, and, during the last winter, to a considerable extent, attacking all the amputations, and nearly all the recent cases. How far any known cause tended to produce it, may, perhaps, be best judged of from a short account of it within the last eighteen months; though, as no accurate observations were made of many concomitant circumstances, much that might have been valuable in regard to it has escaped. In the commencement of the winter of 1837, '38, this disease prevailed to a high degree throughout the whole hospital, both in the medical and surgical wards. Strict measures were taken by Dr. Wallace (then resident) to prevent, if possible, its continuance, such as airing freely the wards, keeping them of a moderate temperature, using but little water in scrubbing, and having all the excretions removed at stated periods. Increased attention to cleanliness was unnecessary, as the institution will at any time advantageously compare with any in the United States in this particular.

These measures, however, had but little effect; the hospital was full, the weather such as prevails generally at the season, and it seemed almost impossible to produce a change in the disease. At last, the whole ward was emptied, scrubbed, white-washed, fumigated, and exposed to the weather for two or three days. The bureaux were searched, and found to contain over a half bushel of musty bread, stale provisions, fruit, &c., which had been concealed, and must have tended to render the air impure. All this was removed, the drawers scalded with lye, and the clothes aired,—after which the ward was reoccupied, and with a decided improvement in every case for some time,—but before the end of the winter the disease returned, but not with its former violence.

In the spring of 1838 it also prevailed slightly, and not generally,—and in the summer and early part of the fall, when the windows were open, and the patients who were able spent much of their time in the grounds, there was hardly a case seen, though there were many bad cases in the hospital, some of which had very free and offensive discharges from the seats of injury.

At the commencement of November the wards were nearly full, especially the men's, and entirely free from it,—but on the 24th, about four weeks after the fires had been kindled, and the weather moist and foggy, it broke out, and continued with intervals of only a week or ten days to the middle of February, notwithstanding the same precautions were used, combined with painting of the bedsteads, tables, &c., a bi-weekly search of the drawers, and the removal of all provisions from the bedside after meals. During this period scarcely a case escaped; one amputation, which had so far recovered as to allow the patient to walk about, sloughed, so as to require a second operation; another terminated in a large abscess considerably above the end of the stump, and after three attacks of the disease it was deemed advisable to send the patient to his friends in the country. A third is still in the house, with the prospect of a tedious exfoliation of near three inches of the tibia, the whole surface having sloughed, and being at present eight weeks since the operation only about two-thirds cicatrized.

Two of the deaths in the fractures were caused by the mortification and debility consequent on attacks of this affection, and two simple fractures were rendered compound from the same cause.

Generally it has terminated almost entirely by gangrene. Of the worst cases, eleven sloughed; one suppurated, and a few terminated in resolution, chiefly in the cases of ulcers and other slight injuries.

At the commencement of an attack there has been always more or less constitutional disturbance, as chill, fever, dry tongue, loss of appetite, irritable pulse, and diminished secretions, with diffused redness more or less marked in the part, heat, swelling, pain and effusion under the skin, after which the redness becomes of a browner hue, matter forms, and the parts around mortify and slough off. In cases of fracture, death of the bone and subsequent exfoliation ensued, leaving the surrounding soft parts in a flabby, unhealthy state, which considerably delayed the cure.

As to the cause, no settled opinion is entertained. That the fires of the furnaces were connected with it seemed probable, until the fact was made known of its prevalence at a former period when the fires were in the wards. Acrid secretions, or large suppurating surfaces, have apparently little influence with us, for, at a time when several extensive injuries were kept in the same room, there was nothing of the kind,—and three amputations were at one period (the last week of February) in the same ward, without any one suffering from it. The most probable cause would seem to be moisture, either external or internal, combined with heat and a collection of animal or vegetable matter, as the only intervals during the season were when the weather was cold and clear, the thermometer ranging from 10° to 25° of Fahrenheit, and a close supervision was kept on the edibles of the patients. It is also offered as a probability, that there is a close connexion between the exciting cause of this disease and the production of miasmata, dependent, in a measure, on the generation, by heat and moisture, of a heavy gas, as the carbonic acid; for, in the room appropriated to the worst cases, where the floor is painted, so as to prevent the absorption of water, and where the ventilator is an opening at the *floor* into the open air, so as to take off the most impure portion of the atmosphere, there is seldom a case as bad as those which prevail in a long ward eighty feet long, with a water-closet in it, and ventilators, as in the rest of the house, opening into the chimnies at the ceilings.

The treatment has mostly been regulated by the state of the case, and the seat of injury. At the commencement, an alterative, followed by a purge, with some mild diaphoretic, were given, and applications made to the part of cold flaxseed mucilage, by means of cloths wrung out of it, and covered with oiled silk, to prevent evaporation; the limb attacked was elevated, to relieve the distension of the vessels, and the part kept at perfect rest by means of a splint.

After this more active measures were resorted to, as the application of leeches to the part, stimulating washes, especially the tinctura saponis camphorata, fermenting poultices of Indian meal and porter, with a full diet, porter, and the free use of quinine. In no case was general bloodletting allowable, and in few cases was the application of leeches necessary,—but, when called for, the American were used around the part, and not the Swedish or Spanish. Under this course, most did well, although several had as many as three attacks successively, in the second and third of which the stimulant plan was used to its fullest extent from the beginning. In some few cases blisters were applied to check or resolve it, but with little success,—and in some there appeared to be rather a tendency to a second attack after their application.

In the use of the soap liniment, which proved the best local application, it was either well rubbed on the part, so as to produce an increased redness in the skin, and quicken the sluggish movement in the capillaries, or it was mixed with the poultice, and applied, warm night and morning.

Incisions were practised in three cases where the disease was attendant on dead bone, and with advantage, by removing the cause of excitement; and when matter collected, it was, of course, freely opened; but scarification, or six or eight incisions into the inflamed and already irritated part, was not practised.

Of the cases treated with quinine, many took it largely, and evidently with benefit; it was pushed in some to doses of thirty or thirty-five grains in the twenty-four hours, but they were patients who were debilitated by the long confinement and the profuse discharge, or by their former habits.

Four cases of *burns* were treated, two of which involved more than two-thirds of the body, and died within twenty-four hours after admission. The others were principally treated by the stimulant practice. One was dressed at first with raw cotton, which was continued for a week, when, on removing the dressings, owing to their offensiveness, several parts were found filled with maggots, produced, no doubt, from the egg having been previously laid in the cotton, as the weather was too cold for the fly to be about. When a severe case is first received, it is sometimes dressed with warm poultices, until the sloughs separate, and is found to be comfortable to the patient, and ready of application.

*Dislocations.*—But two cases of this class of injuries were treated during the winter; one was of the hip, upwards and backwards, of thirteen weeks' standing, in a boy of fifteen years, which, after resisting efforts at reduction of near an hour's duration, was sent out unreduced. The second was of the ulna, upwards and backwards, produced by a fall on the hand, and unattended with fracture. This was reduced, and the man left the house with a good arm.

*Gonorrhœa.*—But five cases of this are marked as admitted, though the number was somewhat greater, as most of these cases are among the sailors, who seldom have syphilis without either a gonorrhœa or gleet, owing to their being taken after leaving port, and not paying much attention to it. The practice pursued, has been free depletion in the acute cases at first, followed by the use of balsam copaiva, and afterwards injections, attention being paid to the state of the alimentary canal. This last had undoubtedly a great influence over the course of the disease, from the strong sympathy which exists between most of the mucous membranes. The use of the copaiva was, therefore, always watched, and as soon as the least symptom of derangement of the stomach appeared, omitted, and some mild purge ordered, to relieve the bowels of it. In the use of injections, the mildest, as a general rule, were prescribed at first, and gradually increased, though, in some cases, after the reduction of the violent inflammation by depletion, purging, and poultices to the penis, a strong injection would frequently stop the discharge, without producing any of the bad symptoms so frequently described as consequences of this mode of treatment, as swelled testicle, stricture and cysticus, a fair opportunity being frequently afforded of judging of the result in cases complicated with buboes, which often detained the patients several weeks in the house. The injections used consisted of acetas plumbi, acetas zinci, sulphas cupri, in the strength of a grain to the ounce, or in argenti nitrat., hydrarg. prot. chlorid. or hydrarg., bi-chlorid., in the proportion of one-third, or a half, or sometimes a grain to the ounce, in the two last always of mucilage of gum arabic. In the treatment of gleets, bougies were always early applied to remove the stricture, so frequently the exciting cause,—after which strong injections, and the use of tonics, especially the tinctura ferri hydriodas, were persevered in.

*Hernia.*—Under this head, are included two cases of an affection of considerable interest, more commonly called *fungus cerebri*, but which are classified here as being deemed more properly herniæ, from the protrusion of the cerebral mass, as proved by dissections, instead of any proper fungus growth. Two cases of this occurred during the season, consequent on fractures of the skull; in one the dura mater was wounded,—in the other it was not, but sloughed, from the protruding of the brain. In both it did not appear till about ten days after the injury, and came on so gradually, as to produce, for some time, very little change in the patients. As the symptoms were similar to the one reported in No. 25 of this journal, it is unnecessary to repeat them. Suffice it to say, that both tended to support the opinion before advanced, of their being the consequences of abscesses near them, and not organized growths.

One case of strangulated femoral hernia, was admitted, and cured by an operation, and is only of special interest, from the long continuance of the strangulation, it having lasted seventy-two hours, and from the peritonitis having extended so far as to cause effusion into the cavity of the abdomen.

*Hæmorrhoids.*—But one case of this was received during the winter, which is now referred to on account of the practice pursued differing from that of many of the profession in this city. Dr. Harris, entertaining the opinion of Richter, Kirby, and others, that the pile is a coagulum of blood formed in the loose, cellular substance, by an exosmosis from the vessels, and covered by the mucous membrane, always removes them by excision, generally by means of a large, curved pair of scissors. Having previously reduced the inflammation of the surrounding parts, the pile is drawn down by a tenaculum, and cut off; and although he has pursued the same practice for a number of years, he has seldom seen hæmorrhage from the operation. In the present case, one of the piles had had a ligature placed around it previous to admission, from which the patient suffered much more than from the operation. After the excision there were not two drachms of blood lost, the bowels were kept quiet for a week, cold applied to the part, and no bad symptoms ensued, and he has since been discharged cured.

*Injuries of joints.*—Few cases are received into the institution, which require such close attention and show so much bad surgery, as this class of affections. Nine cases were treated during the season, several of which were admitted a short time previous to November, all of which offered instances of the result of ignorance on the part of the practicioner. Two cases of injuries to the knee joint may suffice. In one, a man had accidentally run a needle into the knee just below the cap. Unable to find it he continued moving about, applied *lye poultices* and other stimulating applications to it until it was in a terrible state of inflammation, sloughing ensued extending so deep as to expose the capsule of the joint, and subsequent exfoliation of a portion of the tibia, and reducing him so much as to render it doubtful if it would not take his life. Under the treatment pursued a fortunate change took place, and he was discharged cured after being on his back near five months. A second man was received with an incised wound near the inside of the knee—not opening the joint; slight hæmorrhage of course followed—a tourniquet was applied *Tuesday afternoon* and left on till the following *Friady*, the wound partly drawn together and poulticed with lye. The torments of the patient under this treatment were great beyond expression, and on his entrance a week after the injury he had the joint much enlarged, the leg terribly swelled, granulations a half inch high on the wound, and a succession of sloughs under the course of the tourniquet. By perfect rest, soothing applications, &c. he soon became comparatively better, and is now in a fair way of recovery.

The treatment invariably pursued in the house, is one recommended by the late Dr. Physick, of using a carved splint so as to prevent all motion in the part—keeping the patient (if in the lower extremities) on his back—using free purging, vegetable diet, &c., as recommended by him in the treatment of Morbus Coxarius. The same plan is followed in cases of sprains, especially chronic; of fractures near joints, as in the elbow; and universally where there is any risk of inflammation between the articulating surfaces. The splints are carved out of soft wood, so as to receive the limb in the position held at time of entrance, and changed until the desired one is gained; this is worn until all vestiges of inflammation are removed, when motion is gradually commenced. Dr. Randolph invariably follows this course, particularly in violent sprains of the knee, ankle, &c., and in cases of chronic inflammation, has caused them to be worn for twelve or eighteen months, the length of time of course being regulated by the extent of the injury. But one general rule may be pursued, which will mostly produce a successful termination—viz. in no case of injury to a joint to allow of motion, until all symptoms of inflammation are gone; and to do this, not by telling the patient that he must keep perfectly quiet, but to apply some such means as will effectually prevent *all motion* in the part, which can seldom be effected except by the splint spoken of. They can be easily made by any carpenter of common intelligence, by means of a gouge, and a perfect cast of the limb may be taken by applying successive layers of paper and paste to the part desired, or, by marking out the limb on the wood and then scooping it out.

*Operations.*—The number of operations performed during the season has been large, amounting in all to thirty-four, several of which have been capital, and all of which have been successful.

In the *Amputations*, the circular operation was performed in all but one, in which the limb was removed by the catlin. They have been dressed with adhesive plaster, cerate, and a light piece of lint, instead of the old method of a great pledget of tow, which by its warmth too frequently did more harm than good, and have all been healed by granulation.

In that of *Cataract*, breaking and depressing of the lens was practised, sometimes by Saunders' method through the pupil, and sometimes by introducing the knife behind the cornea. The operation had to be repeated several times on one case, but was perfectly successful.

In *Hydrocele* several methods were tried, such as simply evacuating the fluid, and repeating the operation as it reaccumulated; by introducing accupuncture needles and allowing the fluid to escape into the sub-cutaneous cellular substance, but without success; at last the tunica vaginalis was injected with a solution of iodine, as advised by M. Velpeau, which proved effectual. There had been previously a chronic induration of the testis and epididymis, which had resisted the external use of iodine, but which was rapidly changed and improved by the injection.

The *Erectile Tumour*, was of the size of a large walnut on the head of a child, and was cured by the introduction of accupuncturating needles through its base, and surrounding them with ligatures after the manner recommended by Lallemand, and left but a trifling scar.

The *Extirpation of the Parotid Gland* was performed by Dr. Randolph with perfect success, and has been reported at length.

In *Pterygia.*—Dr. Harris performed the operation of dividing the membrane transversely, and applying the nitrate of silver to the cut extremities of the vessels, so as to destroy the growth and nourishment of it, when it is rapidly removed. The objections which he makes to the common operation of excision is, that it interferes with the motion of the eye ball, from the formation of adhesions, preventing the turning of the ball outwards.

In *Varicose Veins* the plan of enclosing the vein by means of a needle passed behind it and a ligature around it was practised, the inflammation consequent on the tightening of the ligatures obliterating the cavity of the vessel. In this case it was practised on both legs at the same time, to prevent the return of old ulcers, and was followed by no bad symptoms.

*Syphilis.*—The cases of this disease which are admitted, are almost exclusively sailors, among whom it generally prevails in its worst form, owing their neglect of its early stages. Eight cases were in the house at the commencement of the season and fourteen have been admitted, all of whom, with two exceptions, have been discharged cured. They consisted generally of numerous chancres, or one or two large ones; in one instance accompanied by phagedenic ulceration of the whole prepuce, condylomatous tumours, or chancres near the anus, with some few cases of syphilitic eruption, and secondary symptoms. The practice pursued has been the *non mercurial* in its fullest extent, not a particle being given, in any case; notwithstanding which, all the cases have rapidly recovered. The treatment has generally been as follows, viz: on the entrance of a patient to order a warm bath, to ensure a perfect cleansing of the parts, then to poultice the penis, and deplete, or purge with saline purgatives if there is much surrounding inflammation. After this is reduced, to make stimulating applications to the chancre, to cause a change of action, as, occasionally, the vegetable caustic, which after the eschar separates leaves a perfectly new, healthy surface, or more frequently the nitrate of silver or sulphate of copper. Cloths wrung out of flaxseed mucilage are then kept to the part to preserve it moist, and it is treated as a simple ulcer. Conjoined to this local treatment, the patient is confined to bed, uses the compound syrup of sarsaparilla freely, and vegetable diet, with attention to the state of his alimentary canal, under which course, few cases of simple chancre require more than ten to eighteen days for their cure. When buboes accompany it, as is frequently the case, they are opened freely as soon as fluctuation can be detected, and afterwards treated by pressure and stimulating washes, perfect rest being always insisted on. When the case, however, is one of secondary syphilis, with eruption, sore throat, nodes, &c., the course is different; purging is freely used and depletion when the pulse demands it, at the same time a daily general bath of warm mucilage is used, with the syrup of sarsaparilla, dulcamara, diaphoretics, &c., when the eruption is obstinate and of long standing, Fowler's solution is freely given, and when accompanied by ulcerated sore throat, the nitrate of silver is applied to the part, or a gargle of the house, consisting of ℞.—Cupri Sulphat. ℈ss.; Pul. Cort. Peruvi. ℥ss.; Pul. Gum. Acaciæ ʒij.; Aquæ fluvial. f℥iv. ft. gargarismus, under which treatment few cases are obstinate. In cases of chancres and tumours at the anus, the pure nitric acid is applied every day or two, and the parts then poulticed, a treatment introduced into the institution, it is believed, by Dr. Randolph. This course seldom gives rise to any of those terrible affections of the bones, &c., so often seen where mercury has been used, and does not, it is thought, render the liability to rheumatism so great. Throughout the year numerous cases are admitted, some presenting the disease in its worst shape, and nearly the same course of treatment is pursued, no one of the surgeons using mercury, and none having to resort to other than the general means mentioned. To the other affections it is deemed unnecessary to refer, as they offer nothing particularly interesting, except in the case of *Sprains.* Of these the treatment was generally perfect rest, with local depletion and cold applications, but in two of those of the ankle, the reverse course was followed, viz. to leech the part very freely immediately after the injury, and then to apply *warm* poultices and perfect rest. Under this plan, the relief is both great and immediate, but further cases are required to test it more fully. In the two spoken of, one of which, was an exceedingly bad one, relief was afforded in thirty-six hours, and the tenderness and swelling of the part was removed in six days, the application of the warmth being spoken of by the patient as very agreeable.

